# 3D culture conditions support Kaposi’s sarcoma herpesvirus (KSHV) maintenance and viral spread in endothelial cells

**DOI:** 10.1007/s00109-020-02020-8

**Published:** 2021-01-23

**Authors:** Tatyana Dubich, Anne Dittrich, Kristine Bousset, Robert Geffers, Guntram Büsche, Mario Köster, Hansjörg Hauser, Thomas F. Schulz, Dagmar Wirth

**Affiliations:** 1 Model Systems for Infection and Immunity, Helmholtz Centre for Infection Research, Braunschweig, Germany; 2 Gynaecology Research Unit, Hannover Medical School, Hannover, Germany; 3 Genome Analytics, Helmholtz Centre for Infection Research, Braunschweig, Germany; 4 Hematopathology Institute of Pathology, Hannover Medical School, Hannover, Germany; 5 Staff Unit Scientific Strategy, Helmholtz Centre for Infection Research, Braunschweig, Germany; 6 Institute of Virology, Hannover Medical School, Hannover, Germany; 7 Cluster of Excellence RESIST (EXC 2155), Hannover Medical School, Hannover, Germany; 8 German Center for Infection Research (DZIF), partner site Hannover-Braunschweig, Hannover, Germany; 9 Institute of Experimental Hematology, Hannover Medical School, Hannover, Germany; 10 Cluster of Excellence REBIRTH (EXC 62), Hannover Medical School, Hannover, Germany

**Keywords:** KSHV infected endothelial cells, 3D culture, Viral maintenance, Episomal viral genomes, Xenograft model

## Abstract

**Abstract:**

Kaposi’s sarcoma–associated herpesvirus (KSHV) is a human tumorigenic virus and the etiological agent of an endothelial tumor (Kaposi’s sarcoma) and two B cell proliferative diseases (primary effusion lymphoma and multicentric Castleman’s disease). While in patients with late stage of Kaposi’s sarcoma the majority of spindle cells are KSHV-infected, viral copies are rapidly lost in vitro, both upon culture of tumor-derived cells or from newly infected endothelial cells. We addressed this discrepancy by investigating a KSHV-infected endothelial cell line in various culture conditions and in tumors of xenografted mice. We show that, in contrast to two-dimensional endothelial cell cultures, KSHV genomes are maintained under 3D cell culture conditions and in vivo. Additionally, an increased rate of newly infected cells was detected in 3D cell culture. Furthermore, we show that the PI3K/Akt/mTOR and ATM/γH2AX pathways are modulated and support an improved KSHV persistence in 3D cell culture. These mechanisms may contribute to the persistence of KSHV in tumor tissue in vivo and provide a novel target for KS specific therapeutic interventions.

**Key messages:**

- In vivo maintenance of episomal KSHV can be mimicked in 3D spheroid cultures
- 3D maintenance of KSHV is associated with an increased de novo infection frequency
- PI3K/Akt/mTOR and ATM/ γH2AX pathways contribute to viral maintenance

**Supplementary Information:**

The online version contains supplementary material available at 10.1007/s00109-020-02020-8.

## Introduction

Herpesviruses exhibit the unique property to establish latent infection for lifelong persistence in the host cells. Kaposi’s sarcoma–associated herpesvirus (KSHV), also known as Human Herpesvirus 8, is a human-specific tumorigenic virus which shares this feature with the rest of the *Herpesviridae* family. The virus infects mainly human endothelial cells, B cells, macrophages, and dendritic cells [1] and it is recognized as the etiological agent of Kaposi’s sarcoma (KS), a tumor of endothelial origin, as well as of two lymphoproliferative disorders: primary effusion lymphoma and multicentric Castleman’s disease [2]. In immunocompetent KSHV-infected individuals, the incidence of KS is low, while in transplant recipients or patients living with human immunodeficiency virus (HIV), it is several hundred to several thousand folds more common than in the general population (reviewed in [3]).

Shortly after infection, the viral genome is circularized and transported into the nucleus of the infected cell, where some viral episomes are tethered to the cellular chromatin [4]. The virus then establishes latency—the viral state characterized by episomal maintenance and a restricted gene expression [5]. KSHV latent nuclear antigen (LANA) is the key protein in latency establishment and viral maintenance; it tethers the KSHV episome with the help of the cellular DNA replication machinery, thereby ensuring duplication of the viral genome during the S phase and accurate segregation of viral genomes during cell division [6, 7].

Occasionally, the virus undergoes lytic reactivation. During this stage of the infection, the majority of viral open reading frames are expressed in a cascade manner, including the viral DNA polymerase, which enables the replication of the viral genome independently of the cellular DNA replication machinery [8–10]. Analysis of KS lesions showed that only a small fraction of the infected cells undergo lytic reactivation, whereas the majority of these cells maintain latency [8].

Histopathology studies and measurements of viral load in KS biopsies showed that although only 10% of endothelial cells are KSHV positive in early lesions of KS, the percentage of the infected cells increases with the progression of the disease, reaching > 90% in late stages [11]. Surprisingly, KSHV-positive spindle cells, isolated from the tumors, tend to lose the KSHV genomes upon cultivation in standard in vitro conditions. Similarly, the majority of newly infected primary endothelial cells, as well as endothelial cell lines, fail to retain the KSHV genome upon cultivation without selection pressure with only a few infrequently arising clones being able to retain the virus without selection pressure [12–17].

We have previously created a conditionally immortalized human endothelial cell line (HuARLT), in which cell growth crucially depends on doxycycline [18]. We demonstrated that these cells maintain the endothelial phenotype upon expansion and closely resemble primary endothelial cells. This includes the expression of key vascular endothelial markers and the ability to take up LDL. Importantly, HuARLT cells are capable of forming vessels in 3D cultures while upon xenotransplantation, they form functional vessels that anastomose with the mouse vasculature, confirming their proximity to primary cells [19]. These cells are susceptible for KSHV infection and establish latency. Latently infected rKSHV-HuARLT cells closely mimic properties described for primary KSHV-infected cells, including spindle-like morphology and transcriptional changes [19]. These changes correspond to endothelial-to-mesenchymal transition, which is observed upon infection of primary cells [20, 21] and is also characteristic for spindle cells isolated from KS [15]. Additionally, rKSHV-HuARLT cells can be cultivated in 3D culture in vitro, where they form spheroids which increase in size upon cellular proliferation [19]. Notably, some KSHV-related features such as the sprouting of endothelial cells are noticeable only under 3D culture conditions [19, 22]. Upon transplantation into mice, these KSHV-infected cells form lesions that exhibit KSHV-dependent growth and that histologically reflect early patch stages of KS [19, 22, 23].

In this study, we took advantage of rKSHV-HuARLT cells to investigate the viral maintenance in endothelial cells. While the virus was lost in 2D culture conditions irrespective of the cellular proliferation status, it was stably maintained in rKSHV-HuARLT cells cultured as 3D spheroids or implanted into immunodeficient mice. We were able to demonstrate a higher reinfection rate in 3D compared with 2D cell culture conditions. We also observed modulations of the PI3K/Akt/mTOR and ATM/γH2AX signaling pathways and used inhibitors of these pathways to show their possible contribution to KSHV maintenance in 3D cell culture conditions.

## Materials and methods

### Cell lines, 2D and 3D culture conditions and treatments

HuARLTs are conditionally immortalized human endothelial cells generated by lentiviral transfer of doxycycline controlled SV40 large T antigen and hTert expression cassettes [18]. For the generation of rKSHV-HuARLT cells, HuARLTs were infected with recombinant KSHV.219 [24] encoding EF1a driven GFP and puromycin selection genes as well as RFP under control of the lytic viral PAN promoter [19]. Puromycin resistant rKSHV-HuARLT cells were established as expandable, doxycycline-dependent long-term cultures. In the presence of puromycin, all cells of the rKSHV-HuARLT cell line express virus-encoded GFP. BFP-HuARLT cells were generated upon lentiviral infection of HuARLT cells with pRRL.PPT.SF.F-EBFP2-F3.pre (kindly provided by Tobias Maetzig, Medical University Hannover, the plasmid backbone was described before [25]) using an MOI of 0.015 and subsequent sorting. If not indicated elsewhere, HuARLT, BFP-HuARLT, and rKSHV-HuARLT cells were cultivated on plates coated with 0.5% gelatin (G1393-100ML, Sigma) in endothelial growth medium (CC-3124, Lonza) in a humidified normoxic atmosphere at 37 °C with 5% CO_2_ and in the presence of 2 μg/ml doxycycline to facilitate proliferation. rKSHV-HuARLT and HuARLT cells show comparable vitalities in the respective culture condition. Maintenance cultures of rKSHV-HuARLT cells additionally contained 5 μg/ml puromycin, while all the experiments were performed in the absence of the selection drug.

The production of 3D aggregates (spheroids) is described in detail elsewhere [19, 20]. In brief, 4000 rKSHV-HuARLT cells per well were first seeded on a 0.5% agarose-coated 96-well plate and cultivated at 37 °C in a humidified normoxic atmosphere with 5% CO_2_ in the presence of 2 μg/ml doxycycline. After 24 or 48 h, spheroids were harvested. Six to 8 spheroids were suspended per 50 μl of 0.7 mg/ml fibrin (341576, Calbiochem), 0.4% methylcellulose (M0512, Sigma), and 0.5 U/ml thrombin (605190-100 U, Merck Millipore) in EGM medium supplemented with 2 μg/ml doxycycline. Matrigel (growth factor reduced, 354,230 Becton Dickinson) was added to the spheroid mixture in a ratio of 1:1. The suspension was cast onto a 96-well plate (100 μl per well) and allowed to solidify for 30 min at 37 °C. One hundred microliters of EGM was added on top of the gels and cultures were maintained in a humidified normoxic atmosphere with 5% CO_2_ in absence of 2 μg/ml doxycycline for the indicated number of days.

For inhibition studies, compounds were tested for concentrations that do not compromise viability. Compound supplemented media was exchanged every 3–4 days in both 2D and 3D culture.

### Viral copy number analysis (qPCR)

DNA was isolated from 2D or 3D rKSHV-HuARLT cell cultures or from Matrigel plugs (see below) according to a protocol described before [22]. For isolation of DNA, cells were lysed using modified Bradley’s buffer with Proteinase K (19133, Qiagen) at 55 °C overnight. Cellular DNA was precipitated by addition of two volumes of 96% ethanol supplemented with 75 mM sodium acetate. After washing with 70% ethanol, the pellet was dried and suspended in nuclease-free water. qPCR was performed at 58 °C annealing temperature using SsoFast™ EvaGreen® Supermix (1725204, Biorad) in a LightCycler 480 II (29376, Roche) using primers specific for viral DNA (LANA) and cellular DNA (ACTB) (supplementary Table 1). The data were analyzed using the Light Cycler 480 software 1.5. For calculation of relative viral numbers, a modified ΔCp method was used, assuming 2 copies of the ACTB gene per cell:

$$ \mathrm{Viral}\ \mathrm{copy}\ \mathrm{number}/\mathrm{cell}=2\ast {2}^{{\mathrm{Cp}}_{\mathrm{LANA}}-{\mathrm{Cp}}_{\mathrm{ACTB}}} $$

### Relative gene expression (RT-qPCR)

A total of 5 × 10^5^ rKSHV-HuARLT cells from 2D or 3D cultures were used to extract RNA using RNAeasy mini kit (74106, Qiagen) according to the manufacturer’s instructions. cDNA was synthetized from 500 ng of RNA using Reverse-Aid First Strand cDNA Synthesis Kit (K1622, Fisher Scientific) according to the manufacturer’s instructions. Quantitative PCR was performed as described above using the primers specified in Supplementary Table 1. Relative expression of viral genes in relation to cellular ACTB was calculated using the standard ΔCp method.

### Flow cytometry

2D cultured cells and 3D spheroids were dissociated with trypsin/EDTA; engrafted cells were isolated as described below. Cells were resuspended in PBS supplemented with 2% FCS. GFP and BFP expression was assessed by flow cytometric analysis (BD™ LSRII analyzer). Non-infected cells served as a negative control. The data were processed with the FlowJo v10 software.

### Immunofluorescence stainings

rKSHV-HuARLT cells were plated on 0.5% gelatin-coated cover glass slips and fixed for 20 min with 4% formaldehyde in PBS followed by permeabilization with 0.5% Triton X-100 in PBS for 10 min. Incubation with PBS supplemented with 2% BSA for 1 h was performed for blocking unspecific binding. The coverslips were stained with primary rabbit anti-Ki67 in PBS with 0.1% saponin Quillaja sp. (S4521, Sigma) at 4 °C overnight. Staining with fluorescently labeled goat antirabbit antibody was performed in PBS with 0.1% saponin at room temperature for 1 h. Coverslips were mounted on glass slides with Fluoroshield™ containing DAPI (F6057-20ML, Sigma) and incubated at room temperature overnight.

For assessing de novo infection of KSHV, infected cells were incubated with primary mouse anti-NCL-HHV8-LNA (Leica Biosystems; 1:100) and stained with a secondary goat anti-mouse Cy5 antibody (Dianova; 1:500). Draq5 staining solution (DR50200, Biostatus) was used for the detection of cellular DNA. Coverslips were additionally incubated with Draq5 (1:1000 in PBS) for 15 min at room temperature and mounted with Mowiol (0713, Carl Roth) overnight.

For immunohistological stainings of engrafted cells, 4 weeks after transplantation of rKSHV-HuARLT cells, Matrigel implants were explanted and fixed with 4% formalin, embedded in paraffine. Sections were stained with hematoxylin-eosin or with antibodies against human vimentin (Dako; 1:100), HHV8 (LANA) (Novocastra/Menarini; 1:30) and GFP (Santa Cruz Biotechnology Inc.; 1:10), using BenchMark Ultra staining machine (Roche). ALU repeats were visualized by in situ hybridization using the ALU probe from Ventana/Roche Diagnostics GmbH and applying Ventana ISH detection kit. Images were acquired using a Zeiss LSM META confocal laser scanning microscope. Brightness and contrast were adjusted using the ImageJ software.

### Transplantation and reisolation of rKSHV-HuARLT cells from mice

rKSHV-HuARLT cells were transplanted to mice as described before [19]. In brief, 1.2 × 10^6^ cells were seeded into each well of AggreWell™400 (27945, Stemcell Technologies), centrifuged for 3 min at 100 g and cultivated for 3 days at 37 °C. Four hundred spheroids were used for each matrigel implant containing 0.2% methylcellulose, 3 mg/ml fibrinogen in EGM media supplemented with 10 μg/ml FGF (100-18B-250, PeproTech), 0.5 μg/ml VEGF (RCPG246, Randox), 1 U/l thrombin (605190-100 U, Merck Millipore), and 300 μl of Matrigel HC (high protein, growth factor reduced, 354263, Becton Dickinson). The mixture was injected subcutaneously to Rag2^−/−^γc^−/−^ mice. 1 mg/ml of doxycycline was added to the drinking water for the whole experiment.

For reisolation of cells from Matrigel plugs isolated from mice, the Matrigel matrix was digested by collagenase H for 1 h at 37 °C. Single-cell suspensions were obtained by cell filtration, using a 40 μm cell strainer. The cells were fixed in 4% formaldehyde for 20 min. In order to discriminate between human and mouse cells, the samples were stained for human Vimentin. To this end, the cells were permeabilized by 0.2% of Triton X-100. The cells were incubated with primary mouse anti-Vimentin antibody (NeoMarkers) for 30 min at 4 °C, washed with PBS, and incubated with goat anti-mouse secondary antibody for 30 min at 4 °C. The samples were analyzed for GFP expression with BD™ LSRII analyzer.

### Gene expression profiling by RNA-Seq

Total RNA was extracted from 2D or 3D rKSHV-HuARLT cultures. Quality and integrity of total RNA were controlled on Agilent Technologies 2100 Bioanalyzer. The RNA sequencing library was generated from 500 ng total RNA using Dynabeads® mRNA DIRECT™ Micro Purification Kit for mRNA purification followed by ScriptSeq v2 RNA-Seq Library Preparation Kit according to manufacturer’s protocols. The libraries were sequenced on Illumina HiSeq2500 using TruSeq SBS Kit v3-HS (50 cycles, single-ended run) with an average of 3 × 10^7^ reads per RNA sample. Each FASTQ file gets a quality report generated by FASTQC tool [26]. Before alignment to reference genome, each sequence in the raw FASTQ files was trimmed on-base call quality and sequencing adapter contamination using Trim Galore! [27] wrapper tool. Reads shorter than 20 bp were removed from FASTQ file. Trimmed reads were aligned to the reference genome using open source short read aligner STAR [28] with settings according to log file. Feature counts [29] were determined using R package Rsubread. Only genes showing counts greater 5 at least two times across all samples were considered for further analysis (data cleansing). Gene annotation was done by R package bioMaRt [30]. Before starting the statistical analysis steps, expression data was log2 transform and normalized according to 50th percentile (quartile normalization using edgeR [31]). Differential gene expression was calculated by R package edgeR. Functional analysis was performed by R package clusterProfiler [32].

### Western blot analysis

On day 3 of cultivation, 3D spheroids were collected and centrifuged; 2D cells were treated with trypsin/EDTA prior to centrifugation. Cell pellets were lysed using ice-cold lysis buffer (50 mM Tris-HCl pH 7.4, 150 mM NaCl, 2 mM EGTA, 2 mM EDTA, 25 mM NaF, 25 mN beta-glyerophosphate, 0.1 mM Sodiumvanadate) supplemented with 0.1 mM PMSF, 5 μg/ml leupeptin, 1 μg/ml aprotinin, 0.2% Triton X100, 0.3% IGEALCA630 (NP40). The pellets were incubated for 30 min followed by centrifugation for 15–25 min at 13 krpm at 4 °C. Cell lysates were transferred to fresh ice-cold tubes. Protein concentration was determined using BioRad photometer with the use of BioRad Protein Assay (5000006, BioRad) according to the manufacturer’s instruction. After addition of 1/5th volume loading dye (21 mM TRIS-HCL, pH.6.8, 21% glycerol, 6,4% SDS, 16% beta-mercaptoethanol, 64 μg/ml bromphenol blue) lysates were boiled, separated on SDS-PAGE, and transferred onto 0.45 μm nitrocellulose membranes (Amersham, GE Healthcare Europe GmbH, or 88018, Thermo Fisher).

Membranes were then incubated with blocking solution (5% non-fat milk (Marvel, Premier Foods, United Kingdom)) in PBS-Tween (0.05%) for 1 h at room temperature and probed with an appropriate primary antibody in blocking solution overnight, at 4 °C. After three times washing, membranes were then incubated with the corresponding horseradish peroxidase (HRP)-conjugated secondary antibodies for an hour at room temperature. After another three times washing, a standard enhanced chemiluminescence (ECL) kit (#34096; Thermo Scientific) or Western Bright Quantum Chemilumineszenz Substrat (541015, Biozym) was used to develop the signals.

The primary antibodies used for Western blot analysis are as follows: RAD50 (ab89, Abcam), MRE11A (GTX70212, GeneTex) NBN (GTX103229, GeneTex), γH2AX S139 (R20244, NSJ Bioreagent), and beta-actin (5441, Sigma). Secondary antibodies are as follows: donkey anti-mouse IgG-HRP (GENA9310, GE Healthcare, now Merck), donkey anti-rabbit IgG-HRP (GENA9340, GE Healthcare).

### Statistical analysis

Statistical significance was determined by *t* test and is indicated by asterisks: *p ≤ 0.05, **p ≤ 0.01, ***p ≤ 0.001, **** *p* ≤ 0.0001.

## Results

### Loss of KSHV genomes in endothelial cell culture is not the result of cell proliferation

**Fig. 1:**
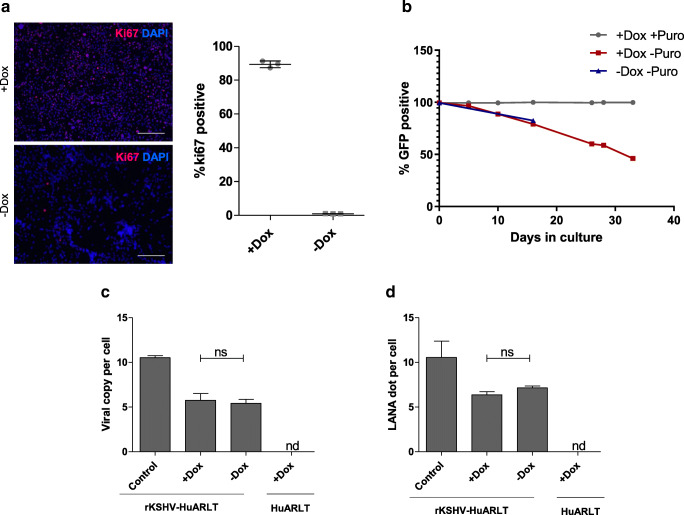
KSHV loss in endothelial cell culture is not the result of cell proliferation **a** To assess the dependence of the proliferation capacity of HuARLT cells on doxycycline, the cells were cultured in the presence or absence of doxycycline for 3 days followed by fixation and the staining with an anti-Ki-67 antibody (red). Nuclei were visualized by DAPI (blue), scale bar 100 μm. Representative images upon immunofluorescence staining are shown from one out of two independent experiments. For quantification, the Ki-67/DAPI ratio was assessed in 3 biological replicates with 3 fields of view per replicate **b** The percentage of GFP positive cells was measured by flow cytometry of rKSHV-HuARLT cells cultured with and without puromycin and doxycycline for up to 33 days as indicated. GFP is encoded by episomal KSHV. The figure depicts one representative experiment out of more than 5 experiments performed **c** The viral copy number of rKSHV-HuARLT cells was assessed by qPCR after 14 days of 2D culture in absence of puromycin and with or without doxycycline as indicated. Data from rKSHV-HuARLT cells cultured in presence of puromycin are shown as a control. Depicted are the results of one out of 4 experiments with 3 biological and 3–4 technical replicates. **d** The viral load was assessed by counting LANA dots per cell upon immunofluorescence staining of rKSHV-HuARLT cells cultured for 14 days in the absence of puromycin and with or without doxycycline as indicated. As control, viral copy numbers of rKSHV-HuARLT cells cultured for 14 days in presence of doxycycline and puromycin are shown. Three replicates were analyzed per condition and 3 independent fields of view were analyzed per replicate. The experiment was performed twice

We evaluated the maintenance of KSHV in human conditionally immortalized endothelial cells, HuARLT cells, which allow strict control of cell proliferation [18]. In the presence of doxycycline, HuARLT cells express the SV40 large T antigen and hTert, permitting cell proliferation, while in the absence of doxycycline, the expression of these genes is switched off leading to the arrest of cell proliferation. In order to evaluate the frequency of proliferating HuARLT cells in the culture, expression of Ki-67, the cellular proliferation marker, was evaluated by immunofluorescence staining of the cells cultured in the presence or absence of doxycycline for 3 days (Fig. 1a). The percentage of Ki-67 positive cells was quantified and the number of Ki-67 positive cells was related to the number of nuclei visualized by DAPI staining per field of view. 89.40 ± 1.16% of Ki-67 positive cells were observed in doxycycline-treated samples, whereas only 0.96 ± 0.06% of the cells were Ki-67 positive upon withdrawal of doxycycline.

To investigate the stability of the KSHV genome upon cultivation of endothelial cells, HuARLT cells were latently infected with rKSHV.219. This recombinant virus confers constitutive GFP expression, the lytic PAN promoter-driven RFP expression as well as constitutive expression of the puromycin resistance gene [24]. Upon propagation in the presence of puromycin, KSHV-infected cells are enriched in culture, eventually achieving a 100% KSHV-positive, latently infected cell population [19, 22]. Similar to non-infected cells, KSHV-infected HuARLT cells maintain the tight proliferation control, as indicated by the comparable cell numbers of infected versus non-infected cultures in absence and presence of doxycycline (Fig. S1). Puromycin resistant rKSHV-HuARLT cells were evaluated for viral stability upon prolonged cultivation in absence of puromycin. To this end, expression of KSHV-encoded GFP was followed by FACS analysis for up to 33 days of cultivation in the presence of doxycycline, or for 16 days in the absence of doxycycline. Note that upon prolonged cultivation, the growth arrested cells undergo senescence [19] and thus cannot be analyzed beyond day 16. The results showed that both proliferating and non-proliferating rKSHV-HuARLT cells exhibited a progressive loss of fluorescence which was accompanied by the appearance of GFP negative cells (Fig. 1b and Fig. S2). This suggests that cells failed to retain the viral copy number upon prolonged cultivation without selection pressure, irrespective of the proliferation status.

Since it was shown that rKSHV GFP expression does not directly correlate with the viral copy number [33], we measured the viral load in proliferating versus non-proliferating rKSHV-HuARLT cells also by qPCR (Fig. 1c) and by counting LANA dots per cell via immunofluorescence, which correlates with viral copy number [34]. The result confirmed a comparable reduction of KSHV copies irrespective of the proliferation status (Fig. 1d). Of note, less than 1% of RFP-positive cells were detected in rKSHV-HuARLT cells under standard cell culture conditions, indicating that a small proportion of cells underwent lytic reactivation irrespective of the culture conditions (Fig. S3). Taken together, the data showed that the proliferation status of the cells did not have an impact on KSHV maintenance, demonstrating that proliferation of cells was not the major cause of viral loss in in vitro cultures.

### rKSHV is maintained in vivo and in 3D culture

**Fig. 2:**
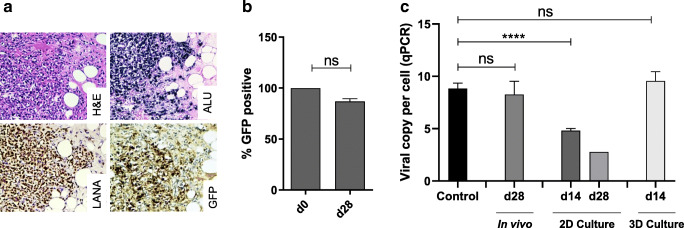
Viral maintenance in vivo and in 3D cell culture. **a** rKSHV-HuARLT cell spheroids were transplanted to RAG^−/−^gC^−/−^ mice. Twenty-eight days after transplantation, plugs were reisolated and stained for hematoxyline and eosine (H&E), for ALU-positive nuclei (ALU), and for GFP and LANA expression. Representative immunohistochemistry sections are shown, magnification × 250. The experiment was performed more than three times. **b** To assess viral maintenance upon transplantation, cells were reisolated from plugs day 28 post transplantation and stained for human vimentin. The frequency of GFP^+^ cells within the vimentin^+^ population was assessed by flow cytometry (d28); it reflects the frequency of KSHV positive cells. An aliquot of the cells before transplantation (d0) is presented as a control. The experiment was performed twice, using 3 mice with two plugs containing 10–20 lesions each. **c** The number of viral copies per cell was determined by qPCR upon isolation from xenograft-transplanted mice on day 28 (d28). The viral copy number was assessed from KSHV-HuARLT cells cultured in 2D and 3D conditions in the presence of doxycycline and in the absence of puromycin for 14 and 28 days as indicated. The viral copy number of KSHV-HuARLT cells in 2D cultures in presence of puromycin is shown as a control. The figure compiles the results of 2 in vivo experiments (3 mice and 1–2 plugs per mouse) and 3 in vitro experiments with 3 replicates. Note that a prolonged cultivation of spheroids in 3D beyond 14 days was not possible because of matrix degradation which compromised stability of the spheroids

Recently, we showed that transplantation of rKSHV-HuARLT cells into immunocompromised mice results in formation of lesions mimicking KS [19, 22]. We asked if there are also signs of viral loss in physiological conditions in vivo. To this end, we subcutaneously transplanted a population of 100% KSHV-positive rKSHV-HuARLT cells in Rag2^−/−^gc^−/−^ mice. An aliquot from the transplanted cells was kept in 2D culture in the presence of puromycin. Twenty-eight days after transplantation, we assessed the expression of GFP and LANA within the lesions via immunohistochemistry. The results showed that the majority of cells within the lesions were positive for both markers of the viral infection (Fig. 2a). In order to confirm this observation, we isolated the human cells on day 28 after transplantation and measured the percentage of GFP-positive cells. The analysis indicated no statistically significant decline in the percentage of GFP expressing cells (Fig. 2b). The viral copy number of reisolated human cells by qPCR was similar to the viral copy number measured before the transplantation (Fig. 2c). This data suggest that—in contrast to the 2D culture conditions in the absence of selection pressure—in vivo conditions support KSHV maintenance in endothelial cells in the absence of puromycin selection. In this regard, the mouse model reflects the situation of infected cells in KS lesions in patients.

We asked if the stability of the virus would be improved upon culturing the cells in a physiologically relevant 3D cell culture system in vitro [19]. To this end, spheroids produced from rKSHV-HuARLT cells were embedded into a Matrigel matrix and cultured in the presence of doxycycline and in the absence of puromycin. Viral copy number analysis showed that cells cultured in 3D conditions maintained the virus copy number for 14 days whereas the control cells in standard 2D culture showed significant viral loss over time (Fig. 2c). This indicates that the conditions supporting KSHV genome maintenance present in engrafted rKSHV-HuARLT cells in mice could be mimicked by 3D culture conditions.

### 3D cell culture conditions support a higher rate of KSHV transmission

**Fig. 3:**
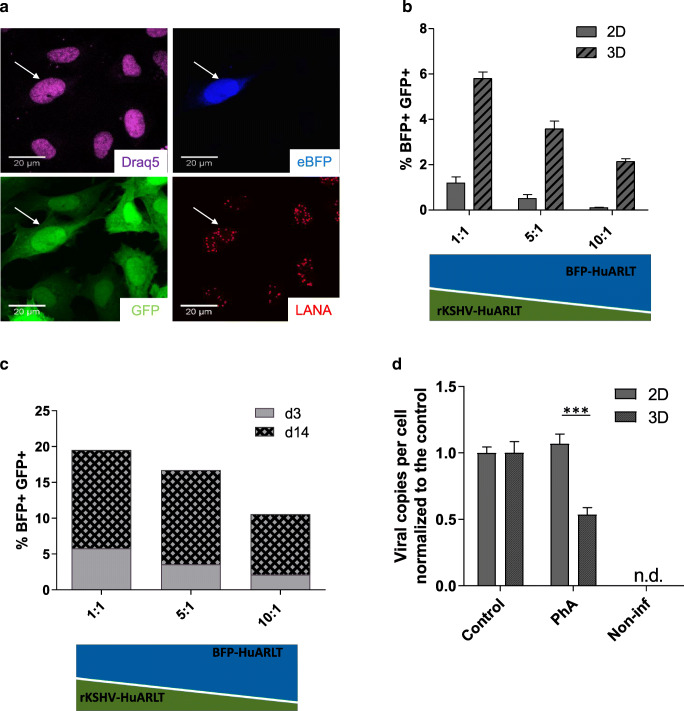
KSHV maintenance in 3D cell culture depends on de novo infection. **a** To assess de novo infection, KSHV-free BFP-HuARLT cells (BFP in the nucleus) were co-cultured with rKSHV-HuARLT cells carrying KSHV-encoded GFP in a ratio of 10:1 for 14 days in 2D in presence of doxycycline and in absence of puromycin. KSHV-infected cells were assessed upon staining for LANA and by monitoring GFP and BFP fluorescence. Draq5 was used to lable nuclear DNA. Fluorescence microscopic pictures using specific filteres are shown for a representative section; scale bar 20 μm. Representative pictures are depicted. **b** KSHV-free BFP-HuARLT were cocultured with rKSHV-HuARLT cells (carrying KSHV-encoded GFP) in indicated ratios in 3D or 2D cell culture for 3 days. The percentage of newly infected BFP^+^GFP^+^ cells out of the BFP^+^ cells was determined by flow cytometry. The figure compiles the results of 4 and 5 independent experiments with technical triplicates for the 2D and 3D conditions, respectively. See Fig. S5 for flow cytometry plots of a representative experiment. **c** The percentage of newly infected BFP + GFP+ cells out of BFP+ cells was determined on day 3 and day 14 of cocultivation in 3D condition based on flow cytometry analysis. See Fig. S5 for flow cytometry plots of a representative experiment. **d** The viral copy number was determined by qPCR upon cultivation of rKSHV-HuARLT cells in 2D and 3D conditions for 14 days in presence of 100 μM phosphonoformic acid (PhA) and normalized to the copy number of cells in the same culture condition in absence of PhA (Control). Non-infected cells (Non-inf) were used as a negative control. n.d., not detected. The graph compiles the data of 2 independent experiments. A representative experiment is shown in Fig. S6

To elucidate the potential mechanism of the differential KSHV maintenance in 2D and 3D cell cultures, we evaluated the frequency of de novo infection. To this end, we established a coculture system that allows the determination of the rate of de novo infection. It consisted of rKSHV-HuARLT cells, which expressed virus-encoded GFP, and non-infected HuARLT cells that were labeled with eBFP (BFP-HuARLT). The cells were mixed together in different ratios and either cultured in standard 2D cell culture or in 3D spheroids for 4 days. In both coculture conditions, BFP^+^/GFP^+^ cells emerged. BFP^+^/GFP^+^ cells were shown to be positive for LANA (Fig. 3a) and carried KSHV genomes (Fig. S4) confirming that these cells were newly infected with KSHV. Importantly, the percentage of newly infected cells was significantly higher in 3D cell culture compared to 2D cell culture and increased with time in culture (Fig. 3b, c and Fig. S5). The higher viral transmission rate in 3D culture conditions might contribute to the improved maintenance of KSHV genomes in the 3D cultures.

### Viral replication contributes to viral maintenance in 3D

To identify mechanisms underlying the differential infection rates in 2D and 3D cell cultures, we assessed the role of KSHV replication and investigated the contribution of the viral DNA polymerase. To this end, we treated the cells with phosphonoformic acid (PhA), an inhibitor of herpesviral DNA polymerase [35]. rKSHV-HuARLT cells were cultured either in 2D or 3D cell culture in the presence of 100 μM PhA and in the absence of puromycin for 14 days, followed by determination of the KSHV copy number per cell. While treatment with PhA did not further decrease the viral copy number in cells cultivated in 2D conditions beyond the decrease due to the absence of puromycin (see Fig. 1), it induced a significant additional loss in viral genome copy numbers in the cells cultivated in 3D (Fig. 3d, Fig. S6). Thus, the data showed that DNA polymerase activity was essential in 3D but not in 2D conditions for maintenance of the viral copy number. Therefore, additional conditions provided by the 3D cultivation might contribute to the improved maintenance of KSHV under these conditions.

### PI3K pathway activation and ATM-mediated gH2AX activation contribute to differential viral maintenance

**Fig. 4:**
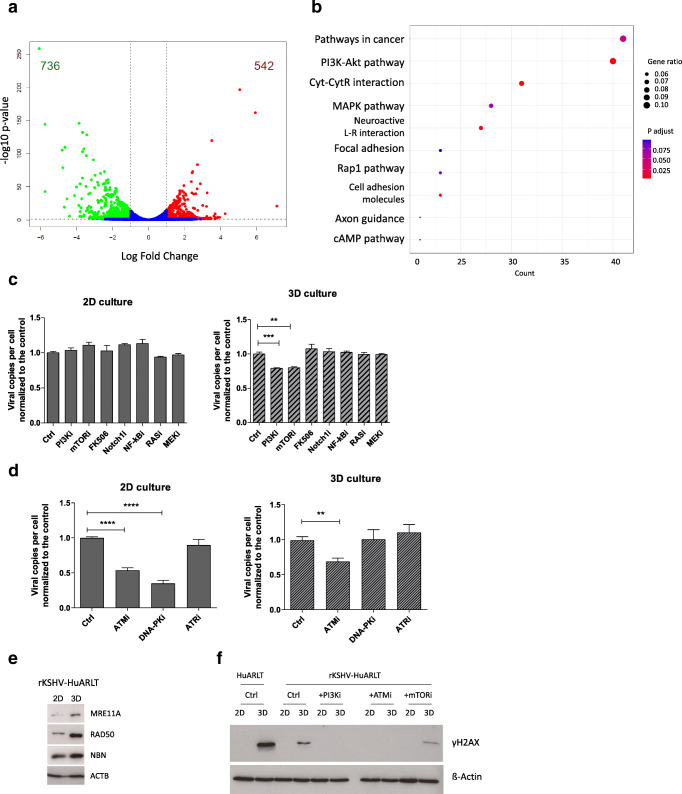
Role of the PI3K/mTOR and ATM/γH2AX pathways in KSHV maintenance. **a** Volcano plot showing differentially expressed genes in rKSHV-HuARLT cells upon cultivation in 2D conditions or in 3D spheroids for 3 days. The genes upregulated in 3D are shown in green; downregulated genes are shown in red. The data are based on one RNA sequencing experiment with two replicates per condition. **b** Top 10 canonical pathways enriched in KEGG pathways by differentially expressed genes from the RNA-Seq analysis of rKSHV-HuARLT cell cultured either in 2D or 3D cell culture for 3 days. **c** rKSHV-HuARLT cells were cultivated in 2D and 3D conditions for 14 days in absence of puromycin and doxycycline and in presence or absence of the following compounds: 2.5 μM LY294002 (PI3Ki), 2.5 μg/ml rapamycin (mTORi), 2.5 μg/ml FK506, 10 μM U0126, 2.5 μM Bay7085 (NF-kBi), 50 μM DAPT, and 5 μM manumycin. Subsequently, the relative viral copy number was measured by qPCR. The viral load in the respective non-treated control samples was set to 1 to account for the regular loss of viral copies during cultivation. Depicted are the compiled results of four independent experiments with 3 biological and 3 technical replicates. See Fig. S8B for a representative experiment. **c** rKSHV-HuARLT cells were cultivated in 2D and 3D conditions for 14 days in absence of puromycin and doxycycline and in presence or absence of the following compounds: 10 μM Ku55933 (ATMi), 1 μM Nu7441 (DNA-PKi), 10 μM mirin (MRNi), or 10 μM VE-821 (ATRi). Subsequently, the relative viral copy number was measured by qPCR. Viral load in the respective non-treated control samples was set to 1 to account for the regular loss of viral copies during cultivation. Depicted are the compiled results of four independent experiments with 3–4 biological and 3 technical replicates. See Fig. S8C for a representative experiment. **d** The abundance of the MRN complex proteins MRE11A, RAD50, and NBN was analyzed by Western blot analysis of rKSHV-HuARLT cells cultured either in 2D conditions or in 3D spheroids for 3 days. A representative blot out of 5 independent experiments is shown. For statistical analysis of RAD50 abundance, see Fig. S9. **e** The presence of γH2AX was analyzed by Western blot of HuARLT and rKSHV-HuARLT cells cultured either in 2D conditions or in 3D spheroids for 3 days (Ctrl). The abundance of γH2AX in rKSHV-HuARLT cells was evaluated in standard conditions (Ctrl) or upon 3d treatment with 2.5 μM LY294002 (PI3Ki), 10 μM Ku55933 (ATMi), and 2.5 μg/ml rapamycin (mTORi). A representative blot out of 2 and 3 independent experiments for 2D and 3D conditions, respectively, is shown

In order to determine which pathways contributed to the differential viral maintenance, we evaluated the cellular gene expression profiles of infected cells in both culture conditions by RNA-Seq. To this end, rKSHV-HuARLT cells were cultured in either standard cell culture conditions or in 3D spheroids for 3 days. Total RNA was extracted and subjected to next generation sequencing. The sequencing reads were mapped to the human reference genome. As a result, 736 cellular genes were found to be upregulated and 542 cellular genes were downregulated upon 3D in comparison to 2D cultivation (Fig. 4a). Differentially regulated genes were strongly enriched in several cellular pathways, among which “Pathways in Cancer,” “PI3K/Akt,” and “MAPK” pathways were the most prominent (Fig. 4b).

Since previous studies showed a critical role of PI3K/mTOR pathway in KSHV pathogenesis during infection, lytic reactivation, and KSHV-induced tumorigenesis, we focused on the cell culture dependent modulation of this pathway. From the 354 genes associated to this pathway, 25 were upregulated, while 15 genes were downregulated under 3D relative to 2D culture conditions (Fig. S7A). qRT-PCR analysis confirmed a moderate but statistically significant upregulation of PI3K, Notch, Hes1, S6K, Jak2, TLR 2/4, and cRaf in 3D culture (Fig. S7B).

Furthermore, to elucidate the relevance of PI3K/mTOR pathway for KSHV maintenance in 3D culture, we specifically blocked PI3K and mTOR activities in virus infected rKSHV-HuARLT cells by small molecules, LY294002 and rapamycin, respectively. To this end, 3D cultures of rKSHV-HuARLT cells were cultured for 14 days in the presence of the small molecules followed by evaluation of viral copy number with qPCR (Fig. 4c, Fig. S8A,B). As a result, treatment with LY294002 and rapamycin led to a statistically significant reduction in the viral copy number in 3D culture of up to 20%, whereas the treatment with FK506, which shares a number of molecular targets with rapamycin, except for mTOR, showed no effect. This observation was confirmed when we used other chemical inhibitors such as IC87114, a specific inhibitor of PI3Kδ inhibitor, and TGX-221, an inhibitor of PI3K p110β (Fig. S8A). In contrast, inhibition of the factors in the PI3K associated Notch, NF-kB, or Ras-cRaf pathways showed no influence on viral copy numbers in 3D culture: when 2D cultures of rKSHV-infected cells were subjected to these inhibitors, the viral copy numbers were not significantly altered compared to non-treated control cells (Fig. 4c). Together, the inhibitor studies indicate a role of the PI3K pathway for viral maintenance in 3D culture conditions.

Perturbation of the PI3K pathway affects many cellular processes such as proliferation, survival, metabolism, differentiation, and also the DNA damage response. The DNA damage response was previously shown to be beneficial for establishment of latency and viral maintenance of KSHV [36, 37]. The DNA damage response is controlled by three kinases, ATM, ATR, and DNA-PK. In order to determine if DNA damage response played a role in KSHV maintenance, we tested if perturbation of this pathway by specific inhibition of the individual kinases would affect the viral copy number in rKSHV-HuARLT cells in the 2D and 3D culture conditions. The cells were treated for 14 days with specific kinase inhibitors followed by the evaluation of the relative viral copy number. No change of copy numbers was observed upon inhibition of ATR in any culture condition while inhibition of DNA-PK only affected copy numbers in 2D culture (Fig. 4d, S8C). A substantial reduction of viral load in 3D culture conditions was only found upon inhibition of ATM, supporting the hypothesis that this pathway is involved in the maintenance of KSHV.

A key regulator of ATM activation is the MRE11A-RAD50-NBN (MRN) complex. Western blot analysis showed some evidence for higher abundance of all three proteins in 3D cell culture of infected cells when related to actin (Fig. 4e, S9). We further analyzed events downstream of ATM in the DNA repair pathway to elucidate the role of ATM for viral maintenance. In particular, we asked if γH2AX, one of the phosphorylation targets of ATM, would be elevated in 3D culture conditions. Western blot analysis also indicated that the amount of γH2AX was higher in 3D cell culture compared to 2D cell culture (Fig. 4f). Moreover, when we blocked PI3K activity by Ly294002 or ATM activity by Ku55933, we could abolish the increase in γH2AX protein expression in 3D cultures of KSHV-infected endothelial cells. Taken together, these data provided evidence for a role of PI3K-dependent, ATM-mediated DNA damage response for KSHV maintenance.

## Discussion

Unlike KSHV-infected B-cells, spindle cells isolated from KS lesions [12–14], as well as newly KSHV-infected HUVECs, [15] fail to maintain viral episomes during cell cultivation in standard 2D conditions. The rapid loss of the KSHV genome upon in vitro cultivation of spindle cells isolated from KS led to the hypothesis that viral loss in endothelial cell culture is a consequence of an imbalance between cell proliferation and inefficient latent replication of KSHV. However, KSHV genomes are efficiently retained in highly proliferative PEL-derived B cell lines, indicating that retention of viral genomes is possible even in rapidly dividing cells. In KS lesions, the proportion of KSHV-infected endothelial cells increases with tumor progression with an overwhelmimg majority of latently infected cells. However, it is unclear, if this reflects the emergence of stably infected subclones with improved viral maintenance in the advanced tumors, or rather the recruitment of uninfected endothelial cells into early KS lesions and a stable new infection rate.

In this paper, we used conditionally immortalized, growth-controlled HuARLT cells [18] to characterize KSHV maintenance in endothelial cells. To this end, we made use of the latently infected rKSHV-HuARLT cells [19, 22], which were generated upon infection of HuARLT cells with the genetically modified rKSHV.219 [24]. These cells were shown to closely mimic properties of primary KSHV-infected endothelial cells in vitro and in vivo [19, 22].

In order to test if differences in proliferation rate contribute to difference in KSHV maintenance, we compared viral copy numbers in proliferating and non-proliferating rKSHV-HuARLT cells. We found no difference in the rate of viral loss in these conditions, showing that the proliferation status was not a deciding factor in the maintenance of KSHV episomes in these cells.

Although rKSHV-HuARLT cells failed to retain KSHV episomes upon prolonged cultivation in the absence of puromycin under standard 2D cell culture conditions, we observed that the majority of the cells within KS-like lesions in mice were GFP-positive and LANA-positive, thus indicating prolonged KSHV maintenance in vivo in the absence of puromycin. The results of the naturally infected rKSHV-HuARLT cells are in line with a previously reported observation based on a BAC-transfected murine cell-based model, which similarly demonstrated loss of KSHV genomes in 2D culture and maintenance in vivo [38]. In order to closely mimic the physiological conditions in vitro, we investigated KSHV maintenance in a 3D cell culture model, in which spheroids of the infected cells were embedded into an extracellular matrix. Interestingly, we observed that the viral copy numbers per cell were maintained in these conditions in the absence of puromycin upon cultivation for at least 14 days.

In order to investigate if the maintenance of KSHV infection in 3D cultures in the absence of selection pressure required de novo infection of cells, we established a highly sensitive coculture model to detect KSHV transmission. Our analysis showed a higher number of newly infected cells in 3D than in 2D cell culture and that the number of newly infected cells increased over the cultivation time.

Similar to other adherent infected cell cultures [39, 40], rKSHV-HuARLT cells are characterized by viral latency and do not show overt lytic reactivation in standard 2D cell culture conditions as indicated by the low number of PAN-RFP-expressing cells. Notably, our newly established co-cultivation system demonstrated low levels of viral transmission in both 2D and 3D culture conditions with significantly higher rates of de novo infection in 3D cell culture. While inhibition of DNA polymerase did not have an effect on the viral copy numbers in 2D cell culture, it strongly affected KSHV maintenance in 3D culture, demonstrating the contribution of lytic reactivation in these conditions. While the particular role of lytic reactivation remains to be elucidated, we hypothesize that another limiting factor may mask the contribution of reactivation for viral maintenance in 2D conditions.

Additionally, viral transmission could be improved by the extended cell-cell contacts in 3D cultures compared to 2D cultures. Previously, direct cell-to-cell transfer of KSHV from B cells to endothelial cells was demonstrated in a 3D co-culture setting [41]. Recent studies showed that also other *Herpesviridae* such as HSV-1 and EBV can spread by direct cell-to-cell contact, thereby evading immune attack, e.g., mediated by neutralizing antibodies. Several pathways have been shown to contribute to cell-to-cell transmission, e.g., exploiting the host endocytic machinery, cell junctions [42], filopodia [43], and tunneling nanotubes [44–46]. Our data indicate that cell-to-cell transfer of KSHV could be possible also between endothelial cells; however, this hypothesis would require further experimental confirmation.

Based on transcriptome analysis using RNA-Seq, the PI3K/Akt pathway emerged as one of the most deregulated pathways in 2D versus 3D cultured KSHV-infected cells. This pathway has been recognized as crucial for various processes during KSHV infection: it is required for viral entry and transport of the viral genome to the nucleus [47], governs survival of the infected cells after establishment of latency [48], and is also necessary for the lytic reactivation of the virus [49, 50]. Moreover, mTOR, one of the downstream targets of the PI3K/Akt pathway, has been shown to promote survival of KSHV-infected lymphatic endothelial cells [51, 52] and treatment of transplant patients with rapamycin, a small molecule inhibitor of mTOR, can induce KS regression [53]. The relevance of the PI3K pathway for lytic reactivation of KSHV was also previously demonstrated upon stimulation of the histamine receptor [54]. Our inhibitor studies thus confirmed the relevance of this pathway for the maintenance of episomal virus genomes in 3D cultures: both, inhibitors of PI3K as well as of mTOR, triggered a notable decrease in the viral copy number. Still, it remains to be elucidated if and to which extend the deregulation of this pathway is only a consequence of viral infection or also contributed by the specific cell culture condition.

Histological studies indicated that markers of DNA damage response, such as γH2AX, pT-Chk2, and 53BP1, are activated in early KSHV lesions [55]. H2AX phosphorylation (γH2AX) can be detected as early as 30-min post-infection in primary endothelial cells and colocalizes with viral DNA [36]. Consistent with the literature data, we observed that inhibition of ATM activity with small molecules led to a significant reduction in KSHV copy numbers both in 2D and 3D cultures. Although we did not detect any deregulation in DNA damage response genes upon transcriptome analysis or qRT-PCR, we observed increased amounts of MRN proteins (MRE11A, RAD50, NBN) upon Western blot analysis in KSHV-infected cells. MRN is a protein complex that mediates recruitment and activation of ATM [56, 57] and, consecutively, phosphorylation of its downstream targets such as H2AX. The MRN complex has also been linked to an innate immune response pathway culminating in the activation of NFkB [58] and KSHV LANA has been reported to interact with components of the MRN complex, thereby modulating the innate immune response and facilitating KSHV reactivation [59]. We could confirm increased phosphorylation of H2AX in 3D cell culture in both non-infected and rKSHV-infected cells. We hypothesize that the activated status of the ATM pathway in 3D cell culture might be a prerequisite for better establishment and/or maintenance of KSHV latency in these conditions.

Several lines of evidence suggest PI3K activation by ATM after DNA damage and as a consequence of insulin signaling (for review, see [60, 61]). Moreover, DNA damage response is modulated by PI3K, e.g., by enhancing in MRE11 expression and stimulating DNA-PK [62–64]. We speculate that both pathways might converge in supporting maintenance of KSHV virus under 3D cell culture conditions.

DNA damage response [65], and in particular, the MRN complex, has also been shown to be critically involved in the maintenance of other viruses such as adenovirus [66], papilloma virus [67], rotavirus [68], and New Castle Disease virus [69]. In addition, MRN proteins were proposed to bind at oriP of EBV and support latent episome replication, probably by resolving recombination like structures [70]. It would be interesting to see in which way the 3D culture mediated changes of cellular properties would also modulate the maintenance of these viruses.

Overall, the observation that endothelial HuARLT cells are able to retain KSHV genomes in vivo and in 3D cell culture conditions while the virus is lost in 2D culture of the same cells paves the way to identify key targets that critically contribute to the control of virus maintenance in 2D vs 3D culture. The identification of such key regulators might thus allow to identify new therapeutic targets to treat KS.

## Supplementary information

ESM 1 (PPTX 4611 kb)

ESM 2 (XLSX 7542 kb)

## Data Availability

The datasets generated during and/or analyzed during the current study are available from the corresponding author on reasonable request. All data and materials, as well as software applications, support their published claims and comply with field standards.
