# Supplementary material for: 3D culture conditions support Kaposi’s sarcoma herpesvirus (KSHV) maintenance and viral spread in endothelial cells
Source: J Mol Med (Berl). 2021 Jan 23;99(3):425–38. doi: 10.1007/s00109-020-02020-8 (PMC7900040; doi:10.1007/s00109-020-02020-8)
Supplement: Supplementary file 1 — (PPTX 4611 kb) [file 109_2020_2020_MOESM1_ESM.pptx]

## Slide 1
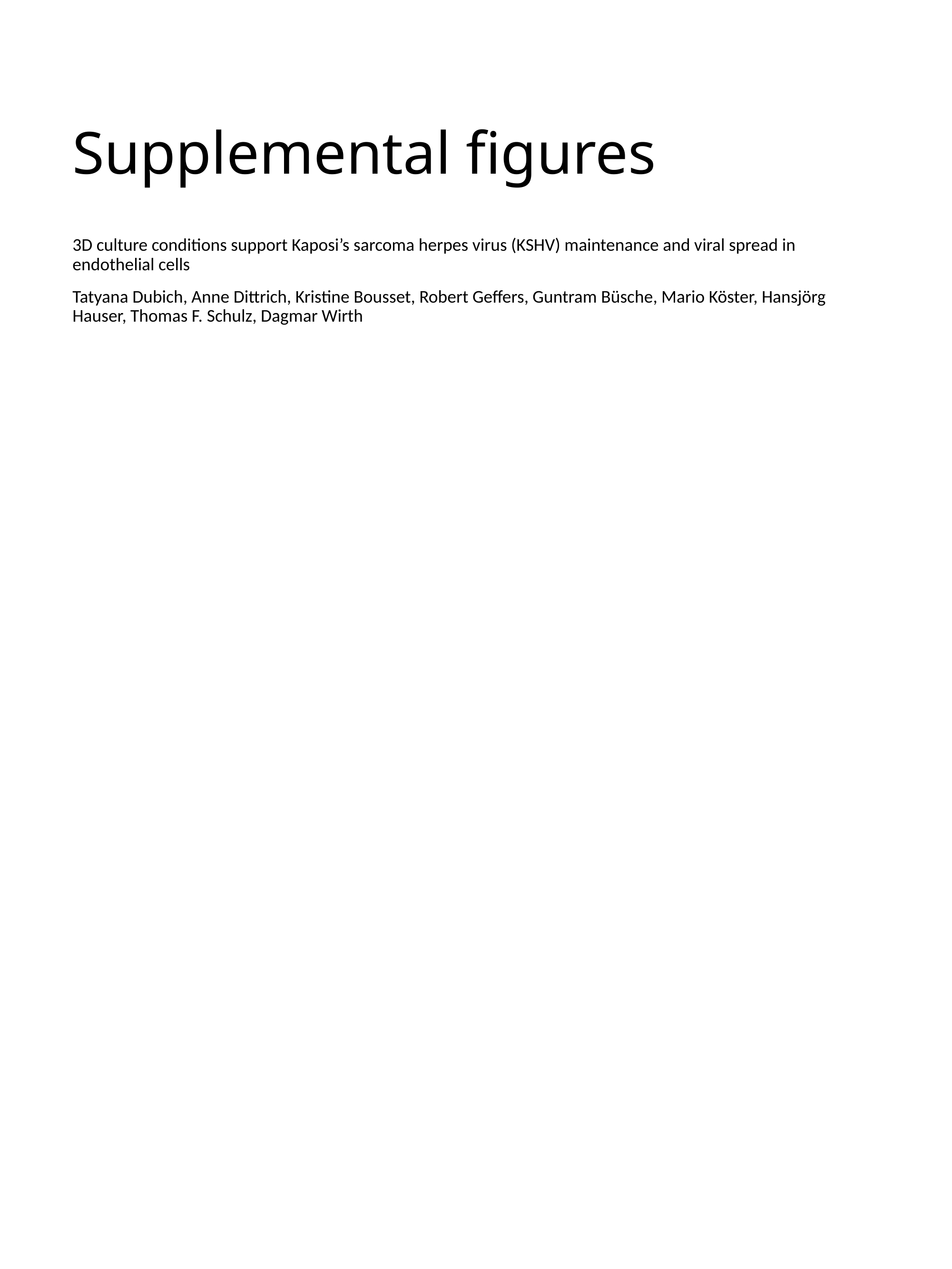

# Supplemental figures
3D culture conditions support Kaposi’s sarcoma herpes virus (KSHV) maintenance and viral spread in endothelial cells
Tatyana Dubich, Anne Dittrich, Kristine Bousset, Robert Geffers, Guntram Büsche, Mario Köster, Hansjörg Hauser, Thomas F. Schulz, Dagmar Wirth

## Slide 2
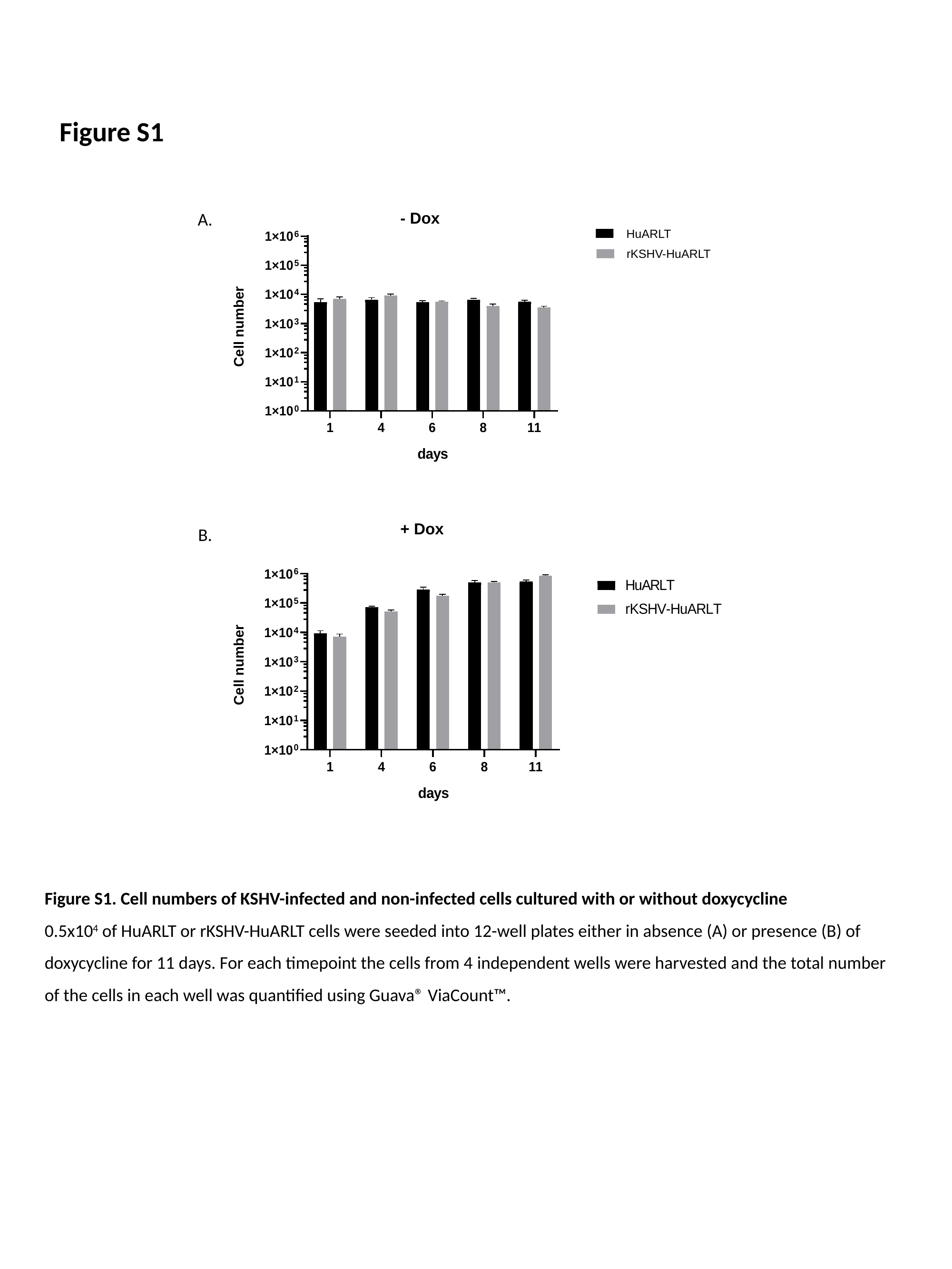

# Figure S1
A.
- Dox
HuARLT
rKSHV-HuARLT
Cell number
+ Dox
B.
Cell number
Figure S1. Cell numbers of KSHV-infected and non-infected cells cultured with or without doxycycline
0.5x10^4^ of HuARLT or rKSHV-HuARLT cells were seeded into 12-well plates either in absence (A) or presence (B) of doxycycline for 11 days. For each timepoint the cells from 4 independent wells were harvested and the total number of the cells in each well was quantified using Guava® ViaCount™.

## Slide 3
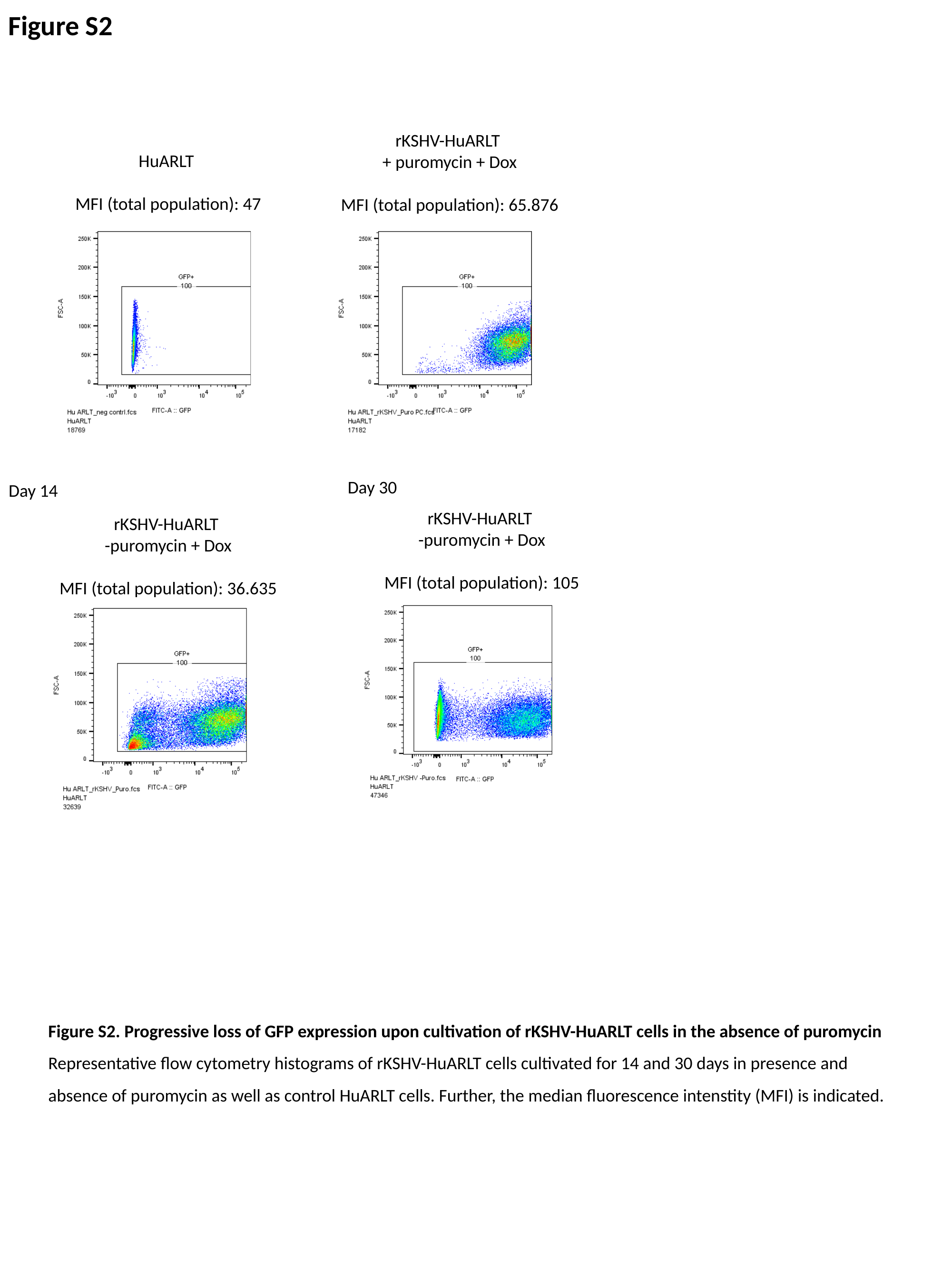

# Figure S2
rKSHV-HuARLT
+ puromycin + Dox
MFI (total population): 65.876
HuARLT
MFI (total population): 47
Day 30
Day 14
rKSHV-HuARLT
-puromycin + Dox
MFI (total population): 105
rKSHV-HuARLT
-puromycin + Dox
MFI (total population): 36.635
Figure S2. Progressive loss of GFP expression upon cultivation of rKSHV-HuARLT cells in the absence of puromycin
Representative flow cytometry histograms of rKSHV-HuARLT cells cultivated for 14 and 30 days in presence and absence of puromycin as well as control HuARLT cells. Further, the median fluorescence intenstity (MFI) is indicated.

## Slide 4
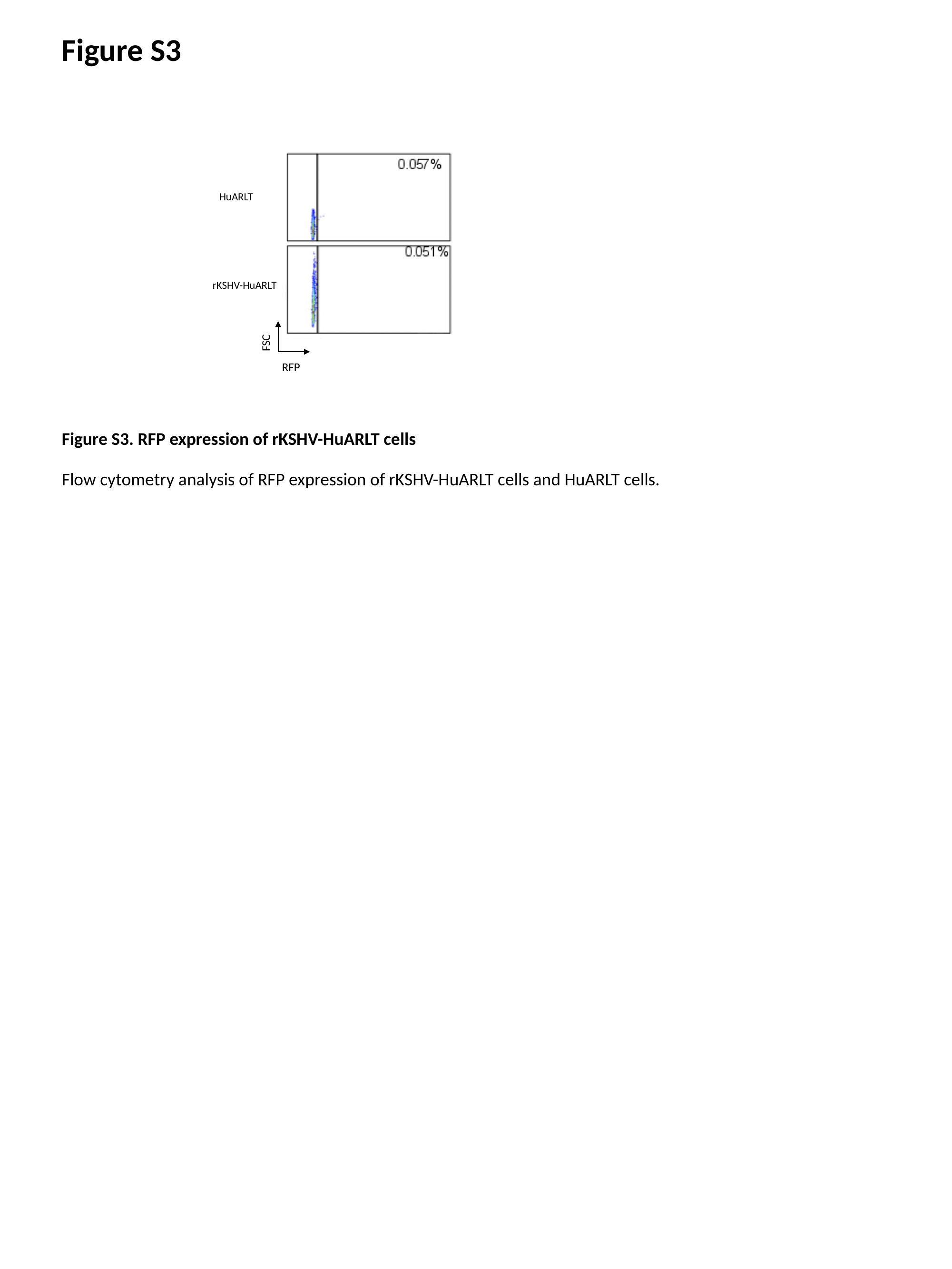

Figure S3
HuARLT
rKSHV-HuARLT
FSC
RFP
Figure S3. RFP expression of rKSHV-HuARLT cells
Flow cytometry analysis of RFP expression of rKSHV-HuARLT cells and HuARLT cells.

## Slide 5
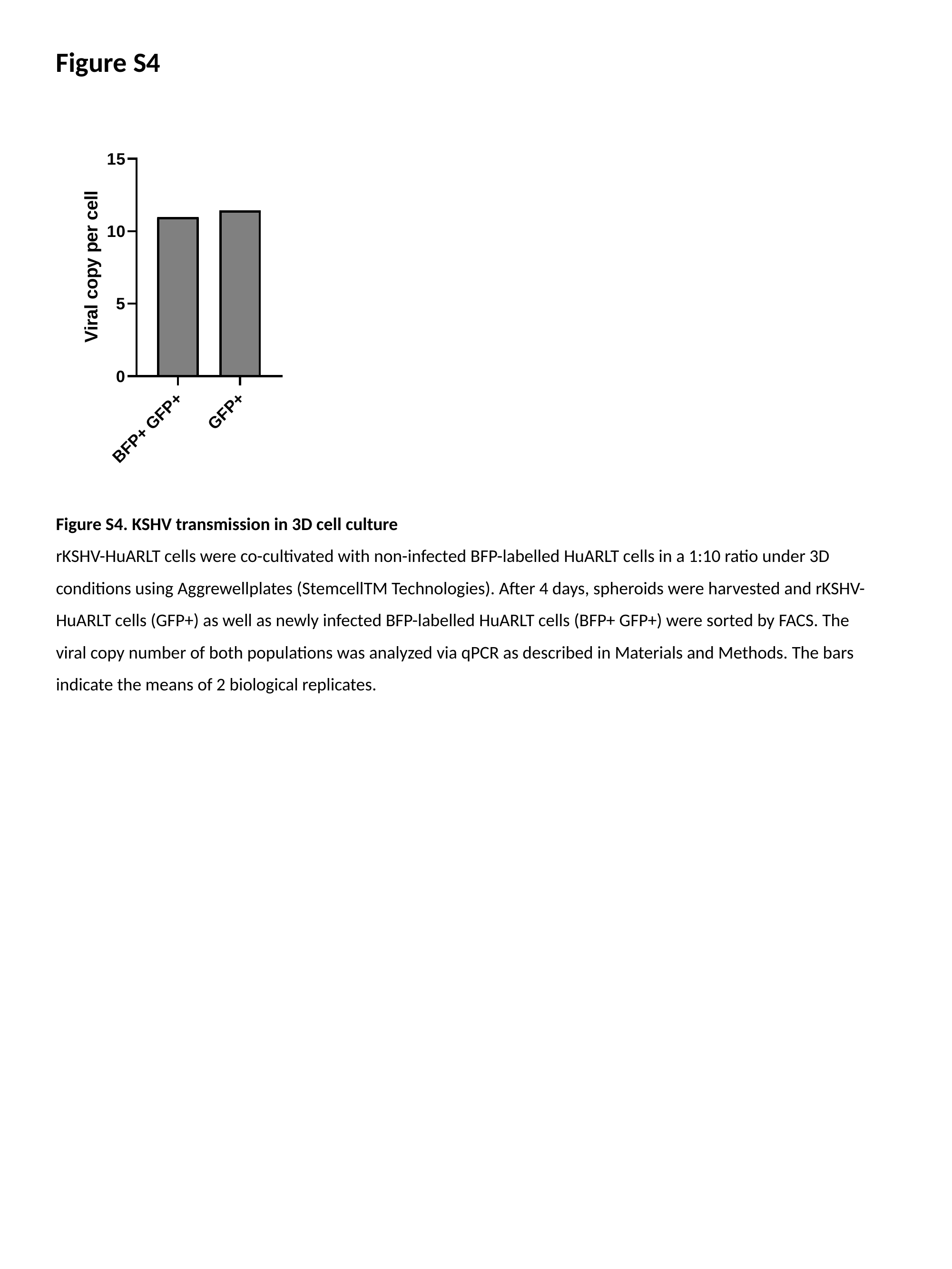

Figure S4
Figure S4. KSHV transmission in 3D cell culture
rKSHV-HuARLT cells were co-cultivated with non-infected BFP-labelled HuARLT cells in a 1:10 ratio under 3D conditions using Aggrewellplates (StemcellTM Technologies). After 4 days, spheroids were harvested and rKSHV-HuARLT cells (GFP+) as well as newly infected BFP-labelled HuARLT cells (BFP+ GFP+) were sorted by FACS. The viral copy number of both populations was analyzed via qPCR as described in Materials and Methods. The bars indicate the means of 2 biological replicates.

## Slide 6
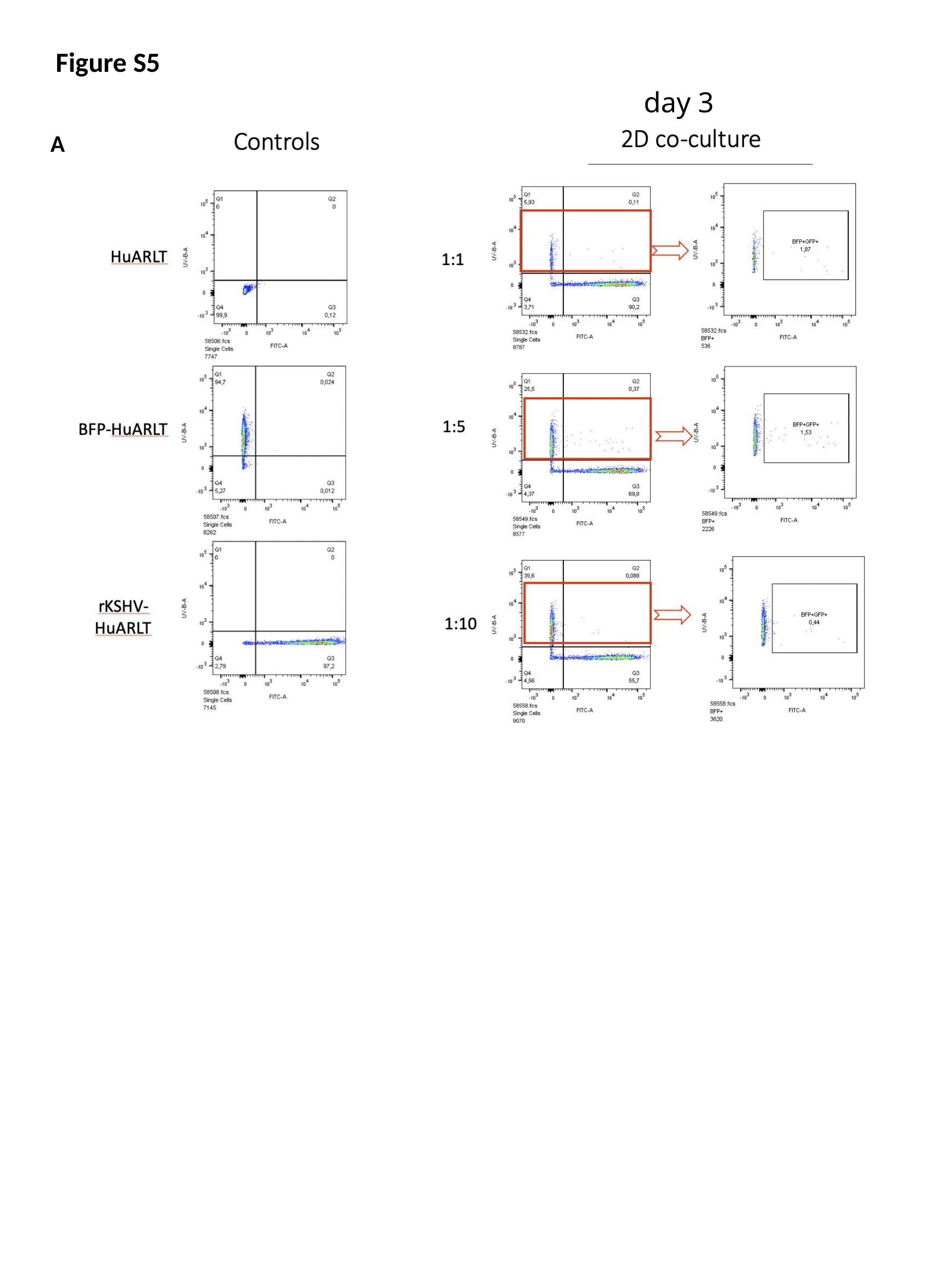

Figure S5
 day 3
A

## Slide 7
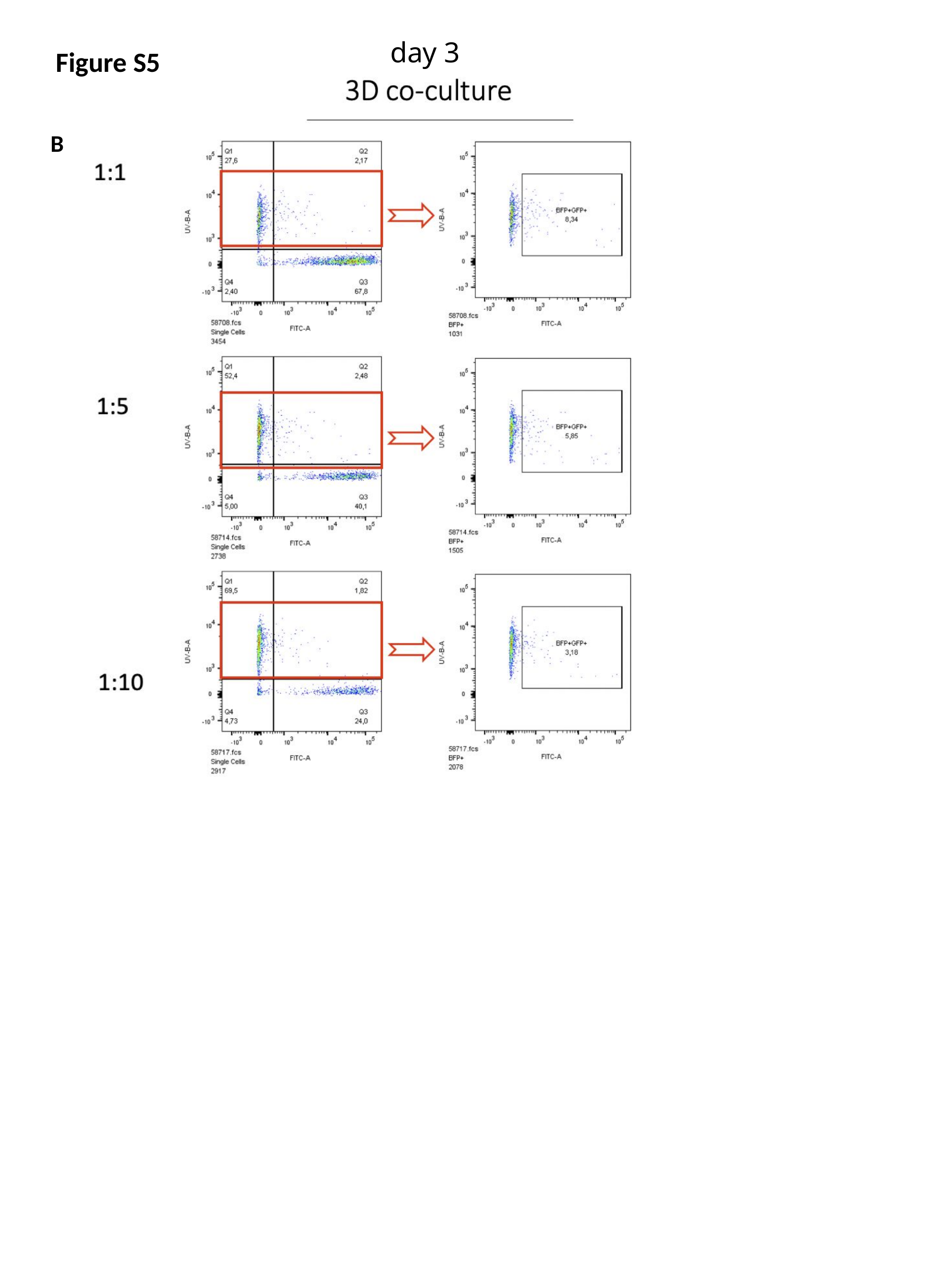

day 3
Figure S5
B

## Slide 8
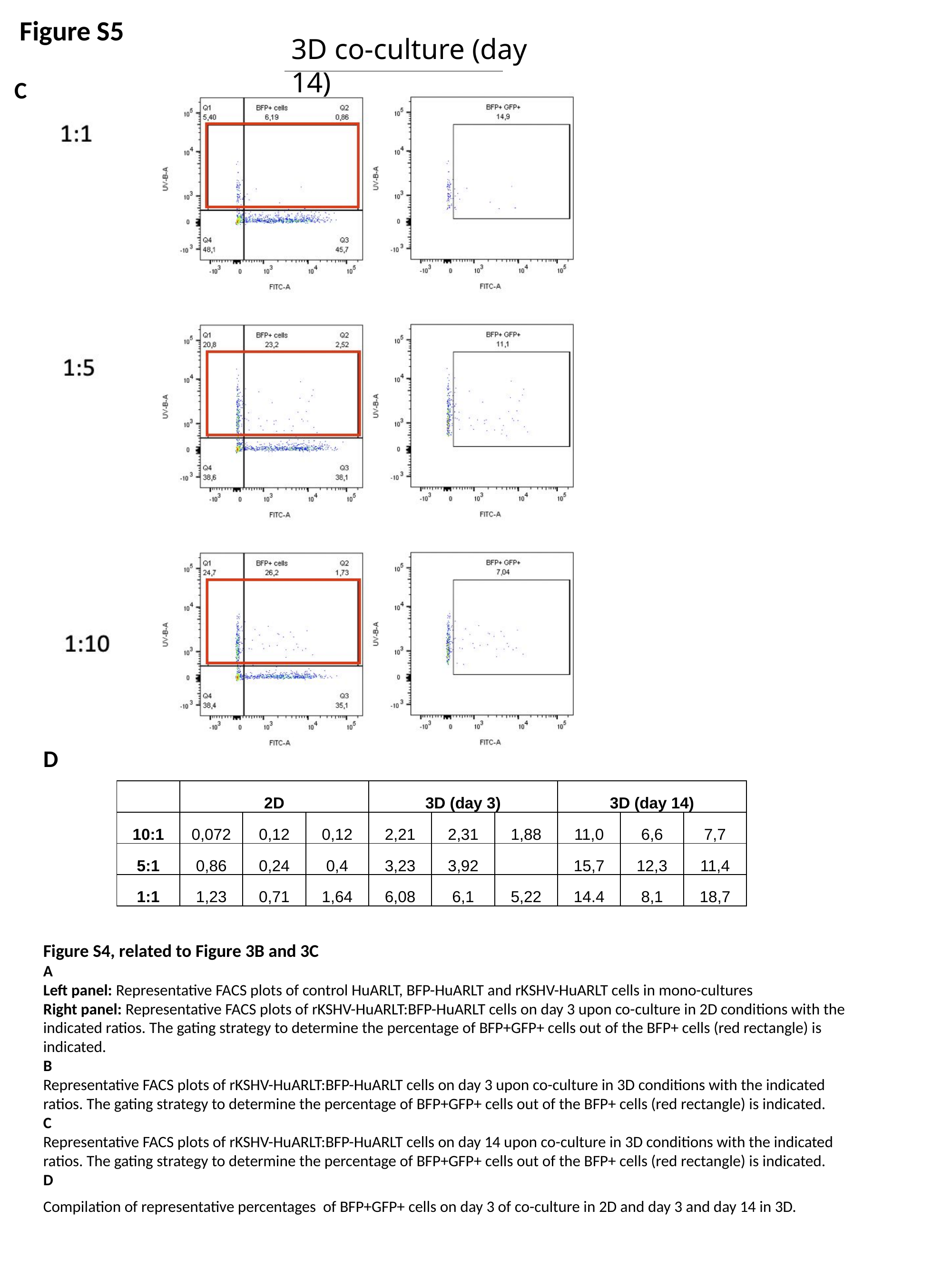

Figure S5
3D co-culture (day 14)
C
D
| | 2D | | | 3D (day 3) | | | 3D (day 14) | | |
| --- | --- | --- | --- | --- | --- | --- | --- | --- | --- |
| 10:1 | 0,072 | 0,12 | 0,12 | 2,21 | 2,31 | 1,88 | 11,0 | 6,6 | 7,7 |
| 5:1 | 0,86 | 0,24 | 0,4 | 3,23 | 3,92 | | 15,7 | 12,3 | 11,4 |
| 1:1 | 1,23 | 0,71 | 1,64 | 6,08 | 6,1 | 5,22 | 14.4 | 8,1 | 18,7 |
Figure S4, related to Figure 3B and 3C
A
Left panel: Representative FACS plots of control HuARLT, BFP-HuARLT and rKSHV-HuARLT cells in mono-cultures
Right panel: Representative FACS plots of rKSHV-HuARLT:BFP-HuARLT cells on day 3 upon co-culture in 2D conditions with the indicated ratios. The gating strategy to determine the percentage of BFP+GFP+ cells out of the BFP+ cells (red rectangle) is indicated.
B
Representative FACS plots of rKSHV-HuARLT:BFP-HuARLT cells on day 3 upon co-culture in 3D conditions with the indicated ratios. The gating strategy to determine the percentage of BFP+GFP+ cells out of the BFP+ cells (red rectangle) is indicated.
C
Representative FACS plots of rKSHV-HuARLT:BFP-HuARLT cells on day 14 upon co-culture in 3D conditions with the indicated ratios. The gating strategy to determine the percentage of BFP+GFP+ cells out of the BFP+ cells (red rectangle) is indicated.
D
Compilation of representative percentages of BFP+GFP+ cells on day 3 of co-culture in 2D and day 3 and day 14 in 3D.

## Slide 9
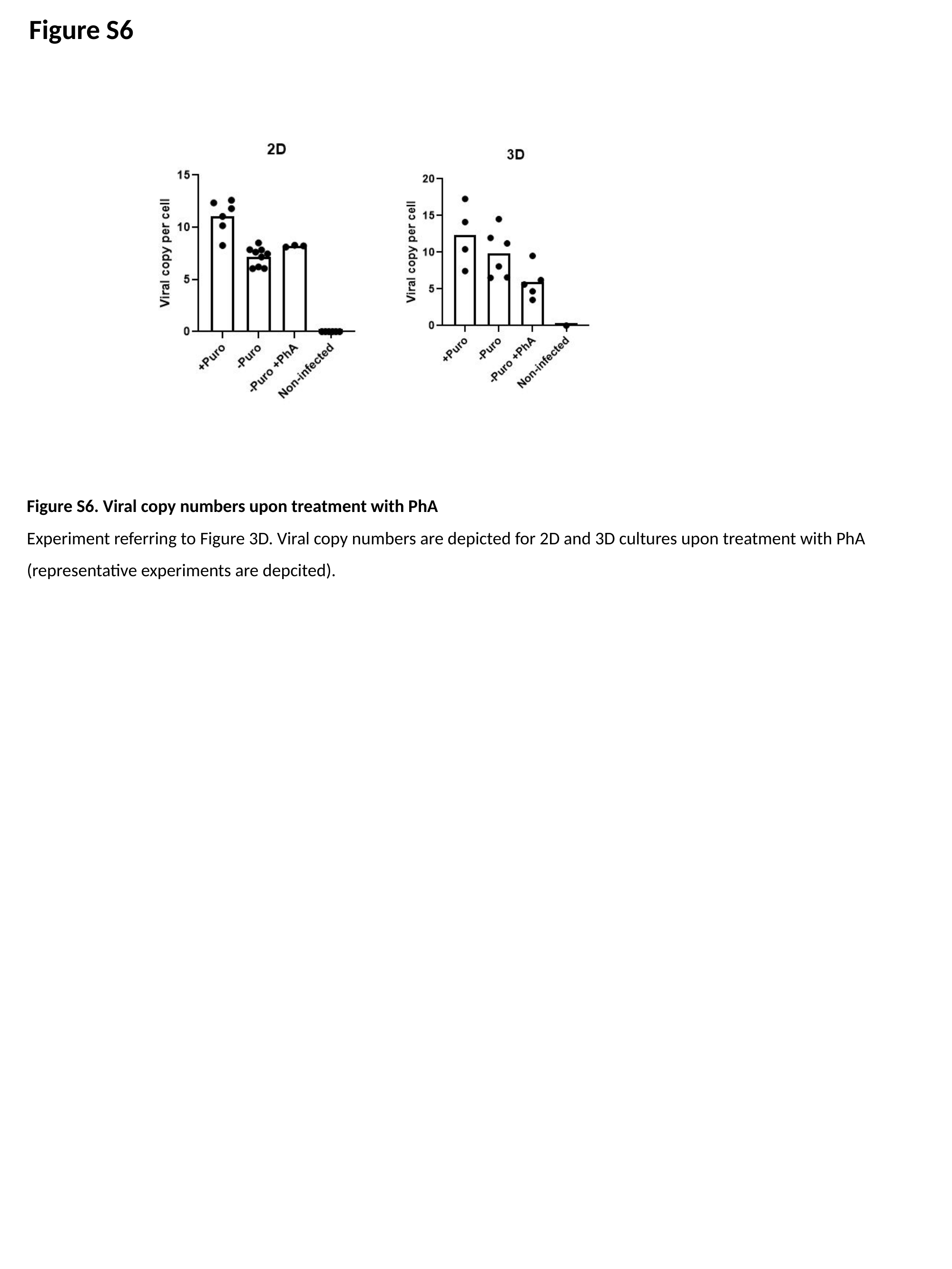

Figure S6
Figure S6. Viral copy numbers upon treatment with PhA
Experiment referring to Figure 3D. Viral copy numbers are depicted for 2D and 3D cultures upon treatment with PhA (representative experiments are depcited).

## Slide 10
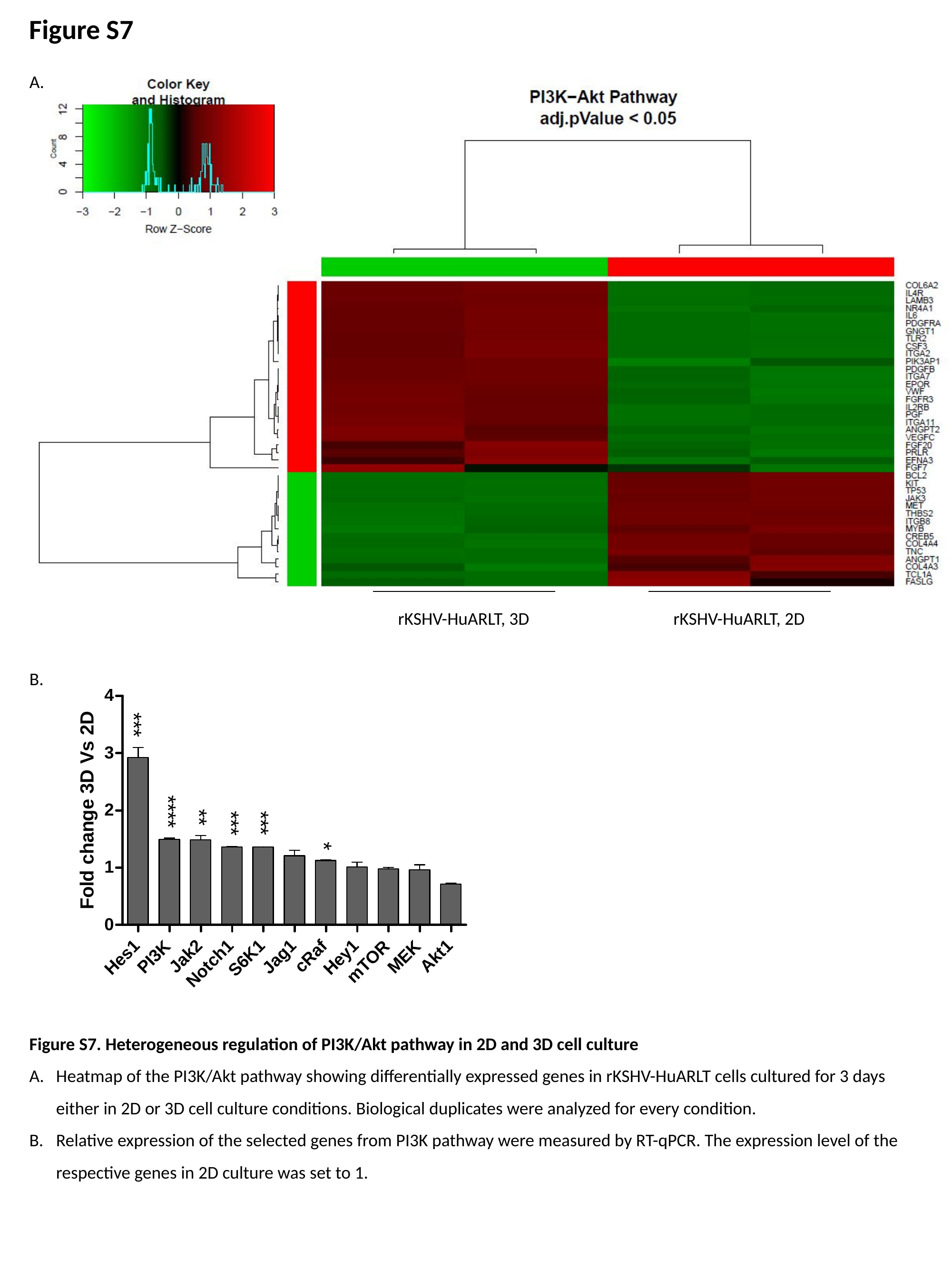

Figure S7
A.
rKSHV-HuARLT, 3D
rKSHV-HuARLT, 2D
B.
Figure S7. Heterogeneous regulation of PI3K/Akt pathway in 2D and 3D cell culture
Heatmap of the PI3K/Akt pathway showing differentially expressed genes in rKSHV-HuARLT cells cultured for 3 days either in 2D or 3D cell culture conditions. Biological duplicates were analyzed for every condition.
Relative expression of the selected genes from PI3K pathway were measured by RT-qPCR. The expression level of the respective genes in 2D culture was set to 1.

## Slide 11
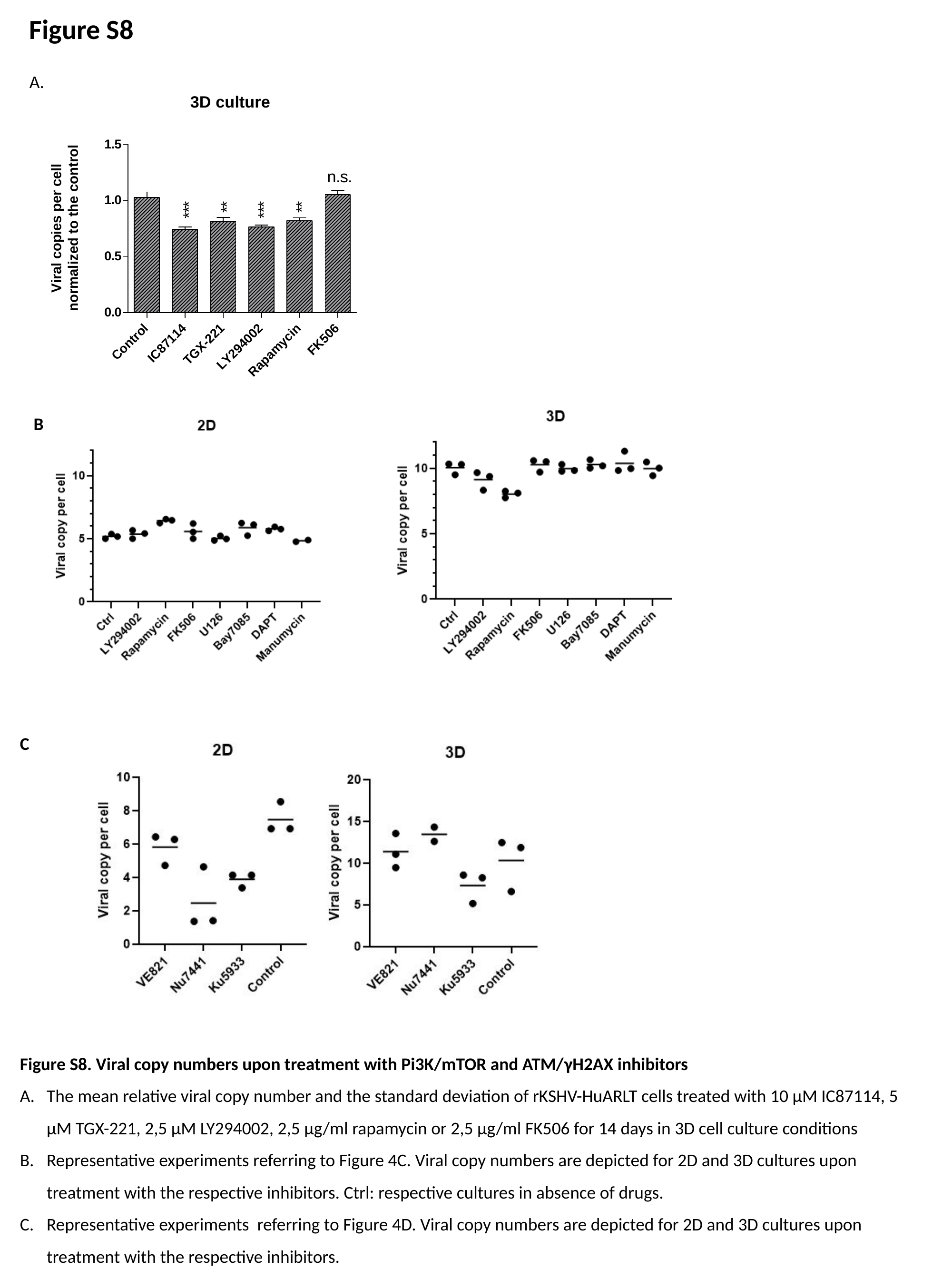

Figure S8
A.
B
C.
C
Figure S8. Viral copy numbers upon treatment with Pi3K/mTOR and ATM/γH2AX inhibitors
The mean relative viral copy number and the standard deviation of rKSHV-HuARLT cells treated with 10 µM IC87114, 5 µM TGX-221, 2,5 µM LY294002, 2,5 µg/ml rapamycin or 2,5 µg/ml FK506 for 14 days in 3D cell culture conditions
Representative experiments referring to Figure 4C. Viral copy numbers are depicted for 2D and 3D cultures upon treatment with the respective inhibitors. Ctrl: respective cultures in absence of drugs.
Representative experiments referring to Figure 4D. Viral copy numbers are depicted for 2D and 3D cultures upon treatment with the respective inhibitors.

## Slide 12
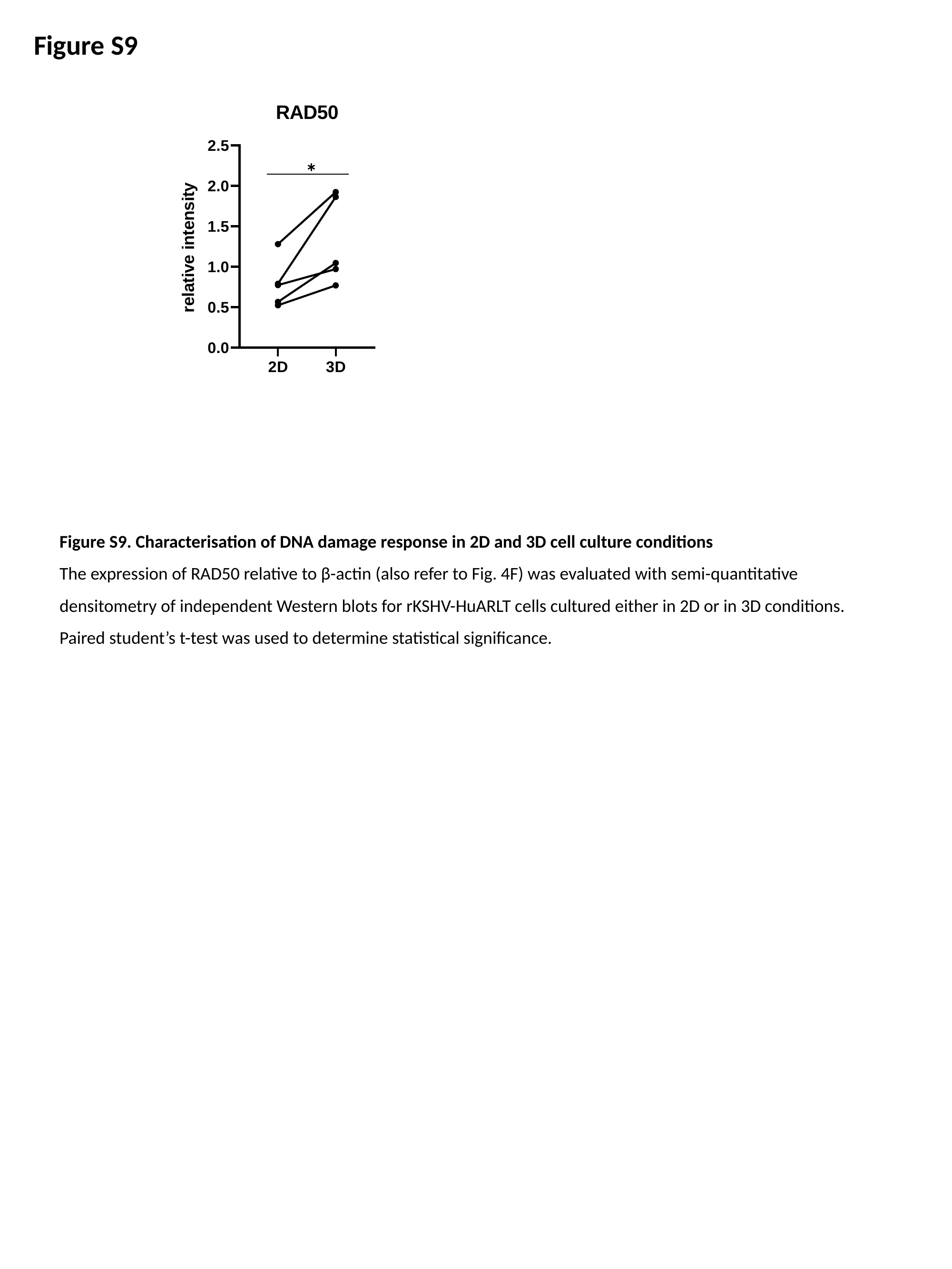

Figure S9
*
Figure S9. Characterisation of DNA damage response in 2D and 3D cell culture conditions
The expression of RAD50 relative to β-actin (also refer to Fig. 4F) was evaluated with semi-quantitative densitometry of independent Western blots for rKSHV-HuARLT cells cultured either in 2D or in 3D conditions. Paired student’s t-test was used to determine statistical significance.
